# Dynamical and Structural Properties of Comb Long-Chain Branched Polymer in Shear Flow

**DOI:** 10.3390/ijms231911290

**Published:** 2022-09-25

**Authors:** Deyin Wang, Xiaohui Wen, Dong Zhang, Jiajun Tang

**Affiliations:** 1 College of Mathematics and Physics, Chengdu University of Technology, Chengdu 610059, China; 2 College of Life Sciences and Institute of Quantitative Biology, Zhejiang University, Hangzhou 310058, China

**Keywords:** multi-particle collision dynamics, dynamical properties, long-chain branched polymer, shear flow

## Abstract

Using hybrid multi-particle collision dynamics (MPCD) and a molecular dynamics (MD) method, we investigate the effect of arms and shear flow on dynamical and structural properties of the comb long-chain branched (LCB) polymer with dense arms. Firstly, we analyze dynamical properties of the LCB polymer by tracking the temporal changes on the end-to-end distance of both backbones and arms as well as the orientations of the backbone in the flow-gradient plane. Simultaneously, the rotation and tumbling behaviors with stable frequencies are observed. In other words, the LCB polymer undergoes a process of periodic stretched–folded–stretched state transition and rotation, whose period is obtained by fitting temporal changes on the orientation to a periodic function. In addition, the impact induced by random and fast motions of arms and the backbone will descend as the shear rate increases. By analyzing the period of rotation behavior of LCB polymers, we find that arms have a function in keeping the LCB polymer’s motion stable. Meanwhile, we find that the rotation period of the LCB polymer is mainly determined by the conformational distribution and the non-shrinkable state of the structure along the velocity-gradient direction. Secondly, structural properties are numerically characterized by the average gyration tensor of the LCB polymer. The changes in gyration are in accordance with the LCB polymer rolling when varying the shear rate. By analyzing the alignment of the LCB polymer and comparing with its linear and star counterparts, we find that the LCB polymer with very long arms, like the corresponding linear chain, has a high speed to reach its configuration expansion limit in the flow direction. However, the comb polymer with shorter arms has stronger resistance on configuration expansion against the imposed flow field. Moreover, with increasing arm length, the comb polymer in shear flow follows change from linear-polymer-like to capsule-like behavior.

## 1. Introduction

Dynamical behaviors, such as tank treading, tumbling and cyclic motions, have been found by many studies for deformable soft polymers under shear flow [1,2,3,4,5,6,7,8]. Such complex motions strongly perturb the flow field through hydrodynamic interactions (HIs) [9,10]. The mixture of both rotational and extensional components [11] in shear flow can cause large conformational changes, in particular for a free polymer [4,5,12,13,14,15,16] and, hence, HIs between the polymer and shear flow fluctuate significantly. Moreover, hydrodynamic simulations have shown that a highly stretched tethered polymer in shear flow reduces the local shear rate remarkably by sticking the surrounding solvent [17,18]. In addition, hydrodynamic simulations have also revealed a non-monotonic stretching response caused by HIs at large shear rates [19,20,21,22]. On the contrary, a study showed that structural and dynamical properties of the semiflexible polymer in shear flow have no dependence on HIs [16]. Theoretical calculations generally neglect such hydrodynamic fluctuations [16,23,24]. To gain more insights into the HIs between polymers and shear flow, there must be more investigations on properties and motions of structural-complex polymers, especially branched polymers.

The properties of the branched polymer in equilibrium and nonequilibrium conditions have been investigated by theoretical analysis [25,26,27,28,29], experiments [24,26,27,30,31,32,33] and simulations [9,34,35,36,37,38,39]. The influence of long-chain branching on the rheological properties of conventional polyolefins, such as polyethylene (PE) [40,41,42,43,44] and polypropylene (PP), has been investigated [45,46,47]. Studies reveal that long-chain branches have a significant impact on the processing properties [48] and have a strong effect on the viscosity as well as the elasticity under small-amplitude oscillatory shear flow [49]. The fundamental characteristics of dynamics of entangled LCB polymers have been explained by the mean-field tube theory, which assumes that the chain is strictly restricted to such a one-dimensional curvilinear tube region, leading to a snake-like repetitive motion [50]. Similar motion that each dangling arm of the LCB polymer performs a repetitive retractive motion starting from its free end all the way down to the junction point on the backbone has also been found [28,29,33]. Nevertheless, the complexity of the structure of LCB polymers (e.g., star, pom-pom, H-shaped, Y-shaped and comb structure, et al.) and interactions among arms leaves the understanding of dynamical behaviors and rheological properties of LCB polymers as still inadequate. Recently, coarse-grained nonequilibrium molecular dynamics (NEMD) studies have shown that a short-chain branched (SCB) linear polymer generally exhibits a more compact and less deformable chain structure. It has reduced shear-thinning and smaller elastic stress behaviors, which are quite distinct from that of the corresponding linear polymer [36,38]. The very short arm of the SCB polymer has a small contact area with shear flow, leading to a high proportion of intrinsic random motions rather than the motions induced by flow in weak-to-intermediate shear flow regime. However, a long arm may experience more regular motions, which are caused by the large contact area in shear flow. Such behaviors of the arm may lead to more rich dynamical behaviors and properties in the LCB polymer. Hence, to explore and understand the influence of branches imposed on the motion and structural fluctuations of the LCB polymer, the study on properties of a comb polymer at a wide range of arm lengths and shear rates is needed.

In this paper, we employ the HOOM-blue molecular dynamics packages to perform the hybrid MPCD and MD simulations [51,52]. The pure Python interface of HOOMD-blue makes the simulation much more adjustable than other traditional simulation software. Therefore, we recommend it and the link of this open-source software is: https://hoomd-blue.readthedocs.io/en/v3.5.0/index.html (accessed on 24 September 2022). We mimic the system by placing a comb LCB polymer in shear flow, which is confined in two parallel planes. During simulation and analyzing processes, we focus on the dynamical and structural properties of LCB comb polymers in shear flow. This work is helpful to understand the transport mechanism of LCB polymers in shear flow and may provide the theoretical guidance for future application in polymer physics and biophysics.

## 2. Results and Discussion

**Figure 1:**
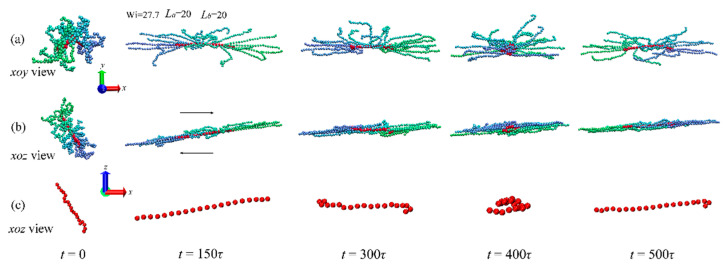
(**a**) *xoy* view and (**b**) *xoz* view of conformations of the LCB polymer with $L_{b}=20,\;L_{a}=20$ in shear flow of Wi=27.7 at various time steps. (**c**) The *xoz* view of the backbone of the above polymer.

In shear flow, the tumbling behaviors are observed for LCB polymers, similar behaviors of linear chains [36], ring polymers [3], short-chain branched (SCB) polymers [36] and star polymers [5] have also been observed. It is shown in Figure 1a,b that a stretched–folded–stretched conformational transition for an LCB polymer under shear flow with Wi=27.7 at *xoy* and *xoz* views, respectively. In addition, the change in arms’ color means that the rotation of LCB polymer contains not only a stretched–folded–stretched conformational transition but also the tumbling behavior of the backbone. The strong stretched state of LCB polymer is maintained; even when the polymer’s backbone at the folded state in the flow-gradient plane (see Figure 1b), such a behavior is the tank-treading motion, which has been observed for the star polymer in shear flow [5]. The *xoy* view of the snapshot (Figure 1a) shows that the LCB polymer’s configuration expands along the flow direction and arms tend to place along the flow direction because the velocity gradient is only present in *xoz* plane. The tumbling behaviors for the backbone are presented in Figure 1c.

**Figure 2:**
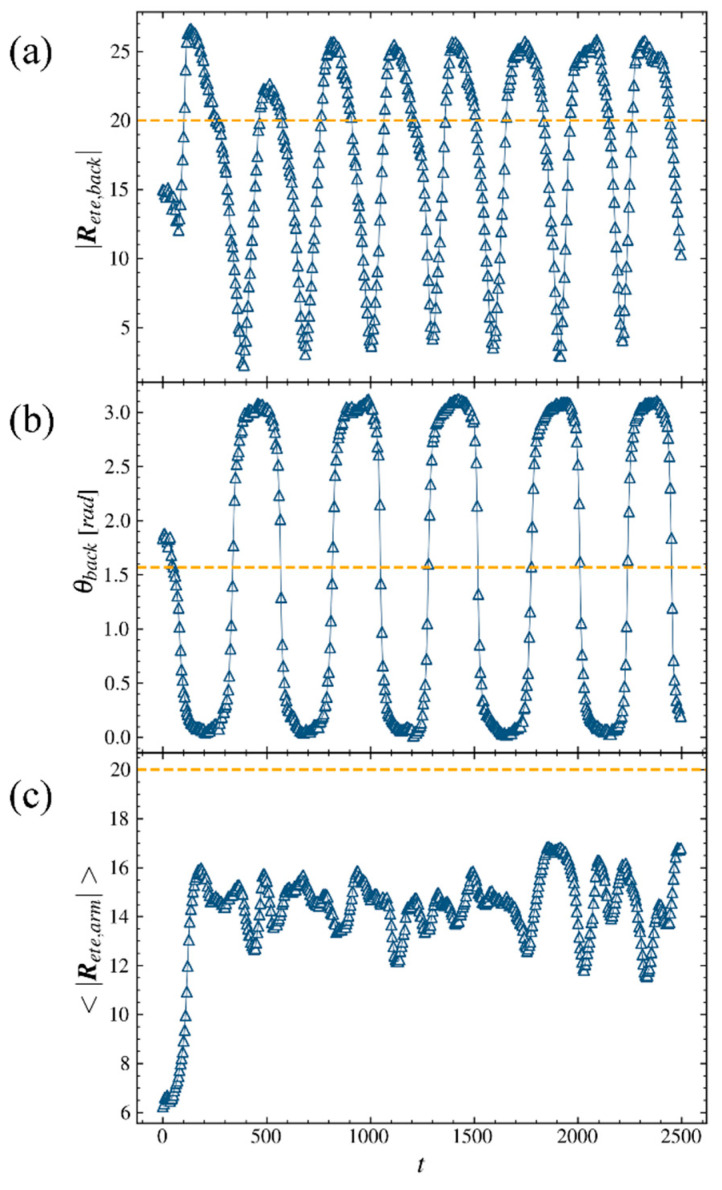
(**a**) The end-to-end distance of the backbone, (**b**) the angle that the backbone’s end-to-end vector makes with respect to the flow direction in *xoz* plane, here the unit of angle is radian. (**c**) The mean end-to-end distances of arms of the LCB polymer are plotted as a function of time. Here $L_{b}=20$, $L_{a}=20$ and Wi=27.7.

To gain more insights into the tumbling and rotation behaviors, we perform a statistic on structural properties of the LCB polymer in shear flow with Weissenberg number Wi=27.7 for time steps. The end-to-end distance of the backbone, the angle that the backbone’s end-to-end vector makes with respect to the flow direction in the flow-gradient plane (*xoz* plane) and the mean end-to-end distance of arms are shown in Figure 2a, b and c, respectively. Figure 2a shows a periodic curve for the end-to-end distance of the backbone; that is, the $\left|R_{ete,back}\right|$ of the polymer increases to a value larger than its contour length $L_{b}=20$ and then gradually decreases to a value smaller than one half of its contour length. The LCB polymer will fold as $\left|R_{ete,back}\right|$ descends and stretch as $\left|R_{ete,back}\right|$ ascends. The low density of points on the ascending line and high density of points on the descending line mean that the folding process is faster than the stretching process in strong shear flow regime. Figure 2a shows the details of stretched–folded–stretched conformational transitions of the polymer. The rotation motion of the polymer and more information of states between two transitions are shown in Figure 2b. In Figure 2b, the angle $\theta_{back}$ periodically changes as simulation time goes on. However, the value is aggregate to 0 or π and then a periodical change occurs between 0 and π at high Weissenberg number Wi. Such a result means that the backbone stays at a strong stretched state and is more likely to remain parallel to the flow direction in strong shear flow regime. The minimum values of $\left|R_{ete,back}\right|$ in Figure 2a correspond to the value of $\theta_{back}$ very close to π/2 in Figure 2b and the stretched–folded–stretched transition corresponds to 0∼π transition of $\theta_{back}$. The average end-to-end distance of the polymer’s arm $\left|R_{ete,arm}\right|$ is very small in the fully relaxed state and increases instantly to a value near 15δ after the shear applies on it (Figure 2c). Non-period fluctuations in $\left|R_{ete,arm}\right|$ displayed in Figure 2c at a long period of time mean that arms of the LCB polymer experience more complex motions because of the effects of entanglement and random motions. However, even under the influence of arms’ disordered motions, the backbone displays periodic stretched–folded–stretched transitions and rotation behaviors. 

**Figure 3:**
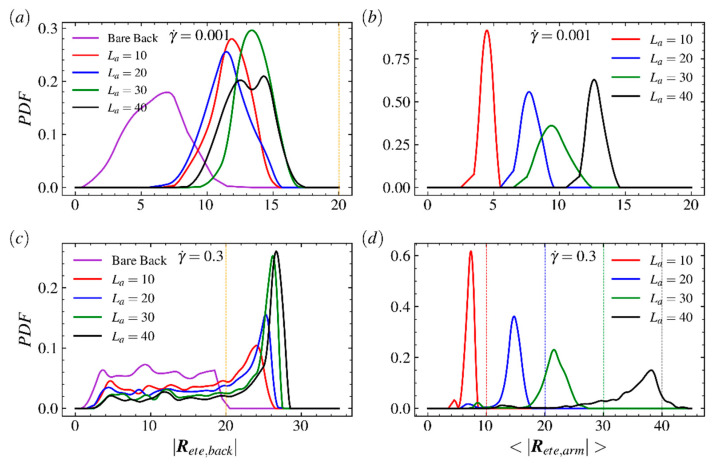
Probability distribution function (PDF) of the end-to-end distance of (**a**,**c**) the backbone and (**b**,**d**) arms of the LCB polymer in shear flow at shear rate $\dot{\gamma }=0.001$ (**a**,**b**) and 0.3 (**b**,**d**). In (**a**) and (**c**), the bare backbone (purple) and comb polymers with arm length $L_{a}=10$ (red), 20 (blue), 30 (green) and 40 (black) are considered. The orange vertical lines in (**a**,**c**) are used to mark the backbone’s contour length $L_{b}=20$. The vertical lines in (**d**) are used to mark the arm’s contour length $L_{a}=10$ (red), 20 (blue), 30 (green) and 40 (black), respectively.

**Figure 4:**
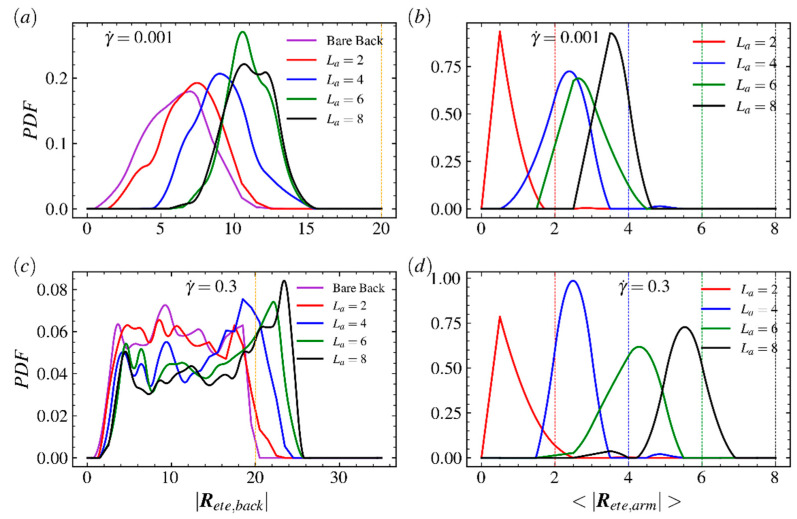
Probability distribution function (PDF) of the end-to-end distance of (**a**,**c**) the backbone and (**b**,**d**) arms of the LCB polymer in shear flow at shear rate $\dot{\gamma }=0.001$ (**a**,**b**) and 0.3 (**b**,**d**). In (**a**) and (**c**), the bare backbone (purple) and comb polymers with arm length $L_{a}=2$ (red), 4 (blue), 6 (green) and 8 (black) are considered. The orange vertical lines in (**a**,**c**) are used to mark the backbone’s contour length $L_{b}=20$. The vertical lines in (**d**) are used to mark the arm’s contour length $L_{a}=2$ (red), 4 (blue), 6 (green) and 8 (black), respectively.

**Figure 5:**
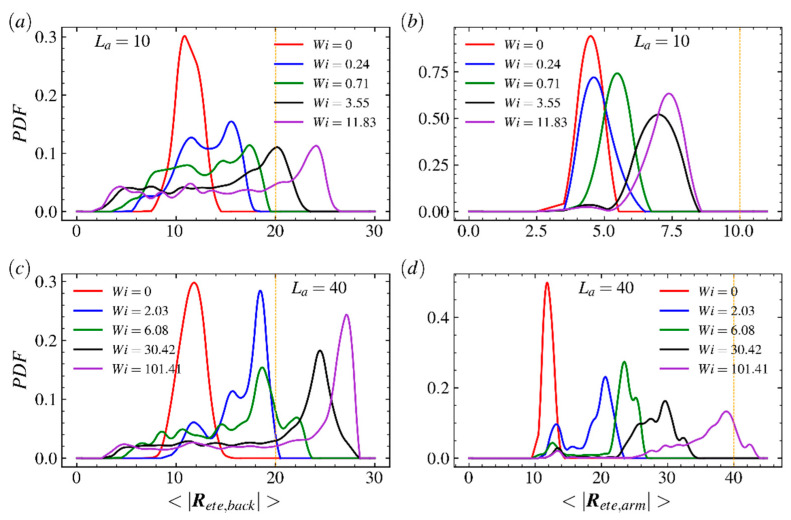
Probability distribution functions (PDFs) of the end-to-end distance of (**a**,**c**) the backbone and (**b**,**d**) arms of the LCB polymer with $L_{b}=20$ and (**a**,**b**) $L_{a}=10$ and (**c**,**d**) $L_{a}=40$ in shear flow with various Weissenberg numbers. The orange vertical lines are used to mark (**a**,**c**) backbone’s and (**b**,**d**) arm’s contour length.

The probability distribution functions (PDFs) of the end-to-end distance of the backbone under weak ($\dot{\gamma }=0.001$) and strong ($\dot{\gamma }=0.3$) shear flows are shown in Figure 3a,c; here, the LCB polymers with arm lengths $L_{a}=10,\;20,\;30,\;40$ and a bare backbone ($L_{a}=0$) are taken into account. The PDFs of the average end-to-end distance of the arm for various LCB polymers are shown in Figure 3b,d; here, the colors of lines correspond to PDFs of backbone in Figure 3a,c, respectively. For the backbone, as shown in Figure 3a,c, the PDFs of the end-to-end distance of the backbone extend and the peak shifts for each LCB polymer and vanishes for the bare backbone as the shear rate increases quickly. In the strong shear flow regime, the peaks of PDFs of the backbone shift toward $\left|R_{ete,back}\right|=30$, which is much longer than its contour length 20, but the extending range of PDF for the bare backbone only reaches backbone’s contour length 20. This is because the strong shear flow decreases the period of stretched–folded–stretched configuration transition and increases the duration of highly stretched states of the backbone. Specifically, the PDF of the bare backbone has an obvious peak in very wake shear flow but the peak range expands and even nearly disappears in strong shear flow regime, indicating linear counterparts of LCB polymers have strong resistance on confined motions against the imposed field. The PDFs of backbones of LCB polymers are adjacent to each other but far from PDF of bare backbone at both small and large shear rate. Meanwhile, in Figure 3c, the peaks increase as the arm length increases. Such results show that, for the LCB polymer, the backbone experiences very strong disturbances imposed by random and flow-induced motions of arms. As the shear strength increases dramatically, the peak positions of these four PDFs are close to its corresponding contour length, implying that most of the LCB polymer’s arms tend to stretch along flow direction at strong shear flow regime and the random motion of the arm has been compressed. For the arm of the LCB polymer, as shown in Figure 3b,d, unlike the position changes on peaks of PDFs of the backbone, which shift to the positions much larger than the contour length of backbone, the positions of peaks for end-to-end distance of arms $\left|R_{ete,arm}\right|$ move toward corresponding contour lengths when shear rate increases from 0.001 to 0.3. It is because arms near the center of the backbone are folded and arms near the ends of the backbone only experience one-side shear. Meanwhile, each arm has one free end and such structure leaves the arm more likely to perform random motions and cannot gain more energy from shear flow field to keep stretching. To gain more insights into the LCB polymer’s response to shear flow, we plot the PDFs of backbone and arms for comb polymers with shorter arms in Figure 4. As shown in Figure 4a,c, the PDFs at high shear rate are closer to each other than that at low shear rate, indicating the random motions of arms play a critical role in weak shear flow regime but the response against applied shear flow becomes critical in strong shear flow regime. As shown in Figure 5b,d, unlike the results for comb polymers with longer arms, the PDFs of polymers with shorter arms shift and extend slightly as shear rate increases, indicating the shear flow is not able to obviously disturb the fast and random motions of shorter arms.

In order to explore the influence of shear flow strength on the LCB polymer’s structure, we depict the probability distribution functions (PDFs) of the backbone length (see Figure 5a,c) and the arm length (see Figure 5b,d) for various Wi. In Figure 5a,c, as Wi increases, the peak of PDF for $L_{a}=10$ decreases and then keeps at a low value near 0.1; however, the peak of PDF for $L_{a}=40$ decreases and then increases. The very different changes mean that the comb polymer with longer arms is more likely to be reshaped by shear flow because the larger configuration span means the comb polymer has more chance to reach the high-velocity flow region. In addition, the strong shear flow makes the polymer shrink to a more compact structure, which means the backbone length has more chance to reach a relatively small value at its folded state, during the stretched–folded–stretched state transition process. Moreover, in Figure 5a,c, the distribution of PDF expands a little when Wi increases from a relatively high value. It is because the impacts imposed by shear nearly reach their limit when Wi increases to a very high value. In Figure 5b,d, similar to PDFs of the backbone length, the peak position of PDF for each arm length shifts to a large value and the distribution broadens as the Wi increases because the large shear rate prolongs the duration of the stretched state and also decreases the duration of structural transition process. However, the ranges of PDFs of arms are not broad like those of backbones because arms have one free end and the arm connecting to the position near two ends of backbone only experience one-side shear. The arm with one free end cannot gain enough energy from the shear flow field to overcome the bond extension energy and keeps its length growing along shear flow direction at intermediate-to-high shear rate. Moreover, the decreasing trend for peaks in Figure 5d means that the long contour length of the arm leaves them far from the center of flow, where the velocities of fluid particles are close to 0 and reach the high shear velocity region. As the arm length increases, the heights of peaks in Figure 5d are much lower than those in Figure 5b, indicating the dynamical behaviors of arms are mainly determined by their contour length.

**Figure 6:**
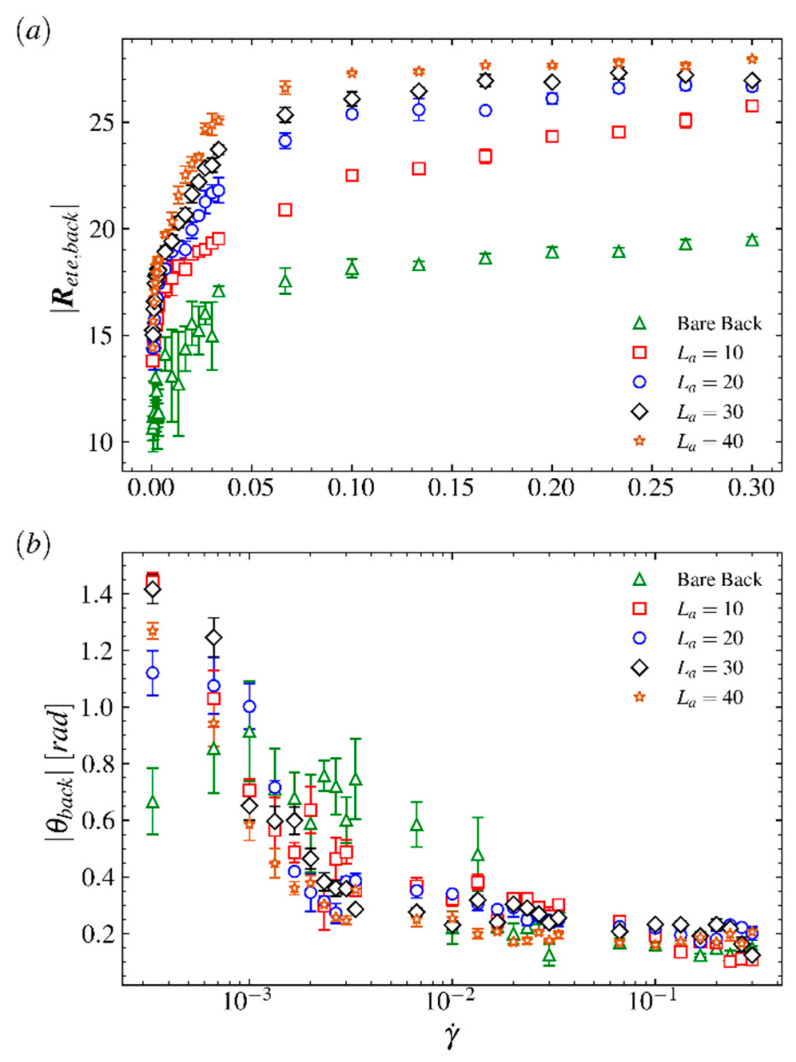
(**a**) The end-to-end distances of the fully stretched backbone for the bare backbone and the LCB polymer as a function of Weissenberg number Wi. (**b**) The angles that the backbone’s end-to-end vectors make with respect to flow direction at flow gradient plane (i.e., *xoz* plane) as a function of Weissenberg number Wi; here the unit of angle is radian.

To explore the mechanism of LCB polymer’s tumbling as the shear flow strength increases, we analyze the end-to-end distance of the backbone $\left|R_{ete,back}\right|$ by varying shear rate and arm length. The end-to-end distances of fully stretched backbones for comb polymers as a function of $\dot{\gamma }$ are shown in Figure 6a, where $L_{b}=20$ and $L_{a}=0$ (bare backbone), 5, 10, 20, 30, 40, respectively. At a wide range of shear rates, due to the random motions and self-entanglement behaviors of long arms $\left|R_{ete,back}\right|$, increases quickly as the arm length increases. It is because the longer arms make the comb polymer more sensitive to strong shear flow and make the backbone’s end-to-end distance reach its upper limit fast as $\dot{\gamma }$ increases. However, the length of the bare backbone at the fully stretched state increases as fast as $L_{a}=40$ but reaches another steady value, which is close to its contour length. This phenomenon indicates that the energy the backbone gains for overcoming elastic force of bonds and stretching longer than its contour length is mainly transferred from arms and determined by the shear flow strength and the arm length. To show the characteristics for the fully stretched state of the comb polymer under shear flow field, we calculate the angle that the end-to-end vector $R_{ete,back}$ of the fully stretched backbone makes with respect to the flow direction and plot it as a function of $\dot{\gamma }$ in Figure 6b. Obviously, the angle $\theta_{back}$ decreases as the $\dot{\gamma }$ increases. It is because LCB polymers tend to stretch strongly along the flow direction and shrink at the velocity-gradient direction in strong shear flow regimes. Specially, in Figure 6b, the value of $\theta_{back}$ for bare backbone is irregular at low shear rate regime but close to the value for LCB polymers at high shear rate regime, indicating that the smaller the configuration span a polymer has, the stronger resistance on structural change against the imposed field the structure has. 

**Figure 7:**
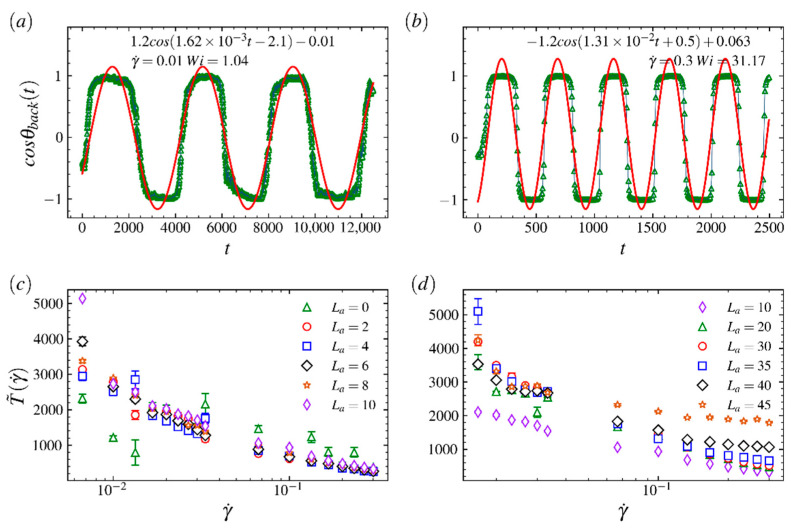
The temporal changes of the cosine value of the angle that the end-to-end vector of the backbone makes with respect to the flow direction at (**a**) Wi=1.04 and (**b**) 31.17, where the backbone and arm length are $L_{b}=20$ and $L_{a}=20$, respectively. (**c**,**d**) The period as function of Weissenberg number Wi for various arm lengths, where the arm length $L_{a}=0$ represents the bare backbone.

**Figure 8:**
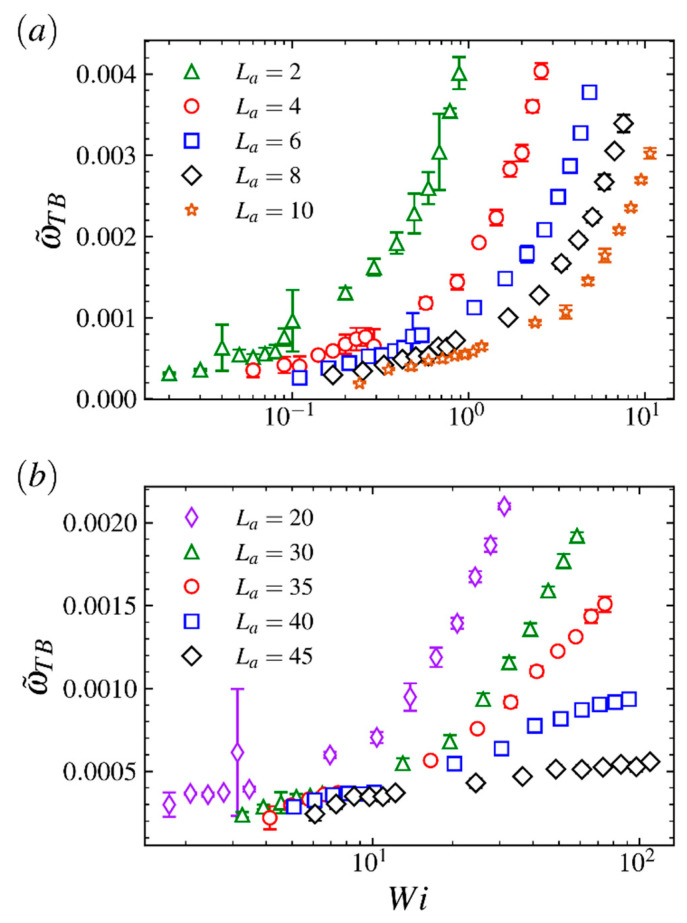
The characteristic tumbling frequency ${\tilde{\omega }}_{TB}$ as a function of the Weissenberg number Wi for comb polymers with (**a**) long arms and (**b**) short arms.

As the stable tumbling and rotation behavior of the LCB polymer is shown in Figure 1a,b, we determine the period of rotation behaviors by fitting the temporal changes of $\cos\theta_{back}\left(t\right)$ to a periodic function: f(t)=Acos(ωt+ϕ)+B. This angle is determined by

(1) $$cos\theta_{back}(t)=<R_{ete,back}(t),\hat{x}>/\left|<R_{ete,back}(t),\hat{x}>\right|,$$

where $R_{ete,back}(t)$ is the end-to-end vector of backbone and $\hat{x}$ represents the unit vector of flow direction. The temporal changes in $cos\theta_{back}$ for Wi=1.04 and 31.17 are plotted in Figure 7a,b, respectively, where the red lines show the results of fitting data with the standard periodic function f(t); here, we choose $L_{a}=20$ and $L_{b}=20$. In Figure 8a,b, the periods of rotations are $2\pi /\left(1.62\times 10^{-3}\right)\approx 3876\tau$ and $2\pi /\left(1.31\times 10^{-2}\right)\approx 480\tau$ for Wi=1.04 and 31.17, respectively. Figure 7c,d plot the period $\tilde{T}(\dot{\gamma })$ as a function of $\dot{\gamma }$ for comb polymers with various arm lengths. In Figure 7c, the rotation period of comb polymers with arms that are shorter than 10 remains adjacent at various shear rates, indicating a gradual configuration expansion induced by small increment on arms will not dramatically influence the rotation behavior of the comb polymer. The irregular changes in period for the bare backbone ($L_{a}=0$) show the very different rotation behaviors between linear chains and comb polymers. The difference indicates that arms have a function of keeping the motion of the branched polymer more stable. Specifically, in Figure 7d, the periods of rotation behaviors of comb polymers with arm lengths $L_{a}=20,\;30,\;40$ are close to each other at small $\dot{\gamma }$ but larger than rotation period for $L_{a}=10$, indicating the comb polymer with long arms has strong resistance, caused by the fast and random motions of arms under medium–weak constraints, to structurally shrink at intermediate to low shear flow regimes. However, the period $\tilde{T}(\dot{\gamma })$ of $L_{a}=20$ and 30 decreases and becomes close to that of $L_{a}=10$ at large $\dot{\gamma }$. Such results mean that random motions of arms have a weak effect on polymer’s structural fluctuations in strong shear flow regime, so the LCB polymer can reach an extremely compact structure. 

In Figure 8, the ensemble averaged tumbling frequency ${\tilde{\omega }}_{TB}$ for the comb polymer with various arm lengths is shown as a function of Weissenberg number Wi. As shown in Figure 8a, for the comb polymer with relatively short arms, the curve for characteristic tumbling frequency keeps a similar exponential increasing trend as the arm length $L_{a}$ increases from 2 to 10. Such obvious exponential dependence of the tumbling frequency on Weissenberg number has been observed for ring and star polymers [38,53,54]. However, for comb polymers with relatively long arms (see Figure 8b), the exponential growth of ${\tilde{\omega }}_{TB}$ decays to linear as the arm length grows further from 35. Similar behaviors for capsules [55] and star polymers [5] have been observed in strong shear flow regime. Such results indicate that the LCB polymer with very long arms is likely to have properties similar to the capsule at large Wi and has high resistance on massive shape fluctuations. The different changes in curves from varying the arm length from small and large value also indicate that the polymer size plays a critical role in the dynamical properties when the comb polymer is placed in shear flow. Meanwhile, as shown in Figure 8b, the value of ${\tilde{\omega }}_{TB}$ decreases significantly as the arm length grows from 30 to 45 in the strong flow regime, implying that the comb polymer with very long arms has properties that are similar to the star polymer and the capsule, distinct from the comb polymer with small-to-intermediate branch size.

**Figure 9:**
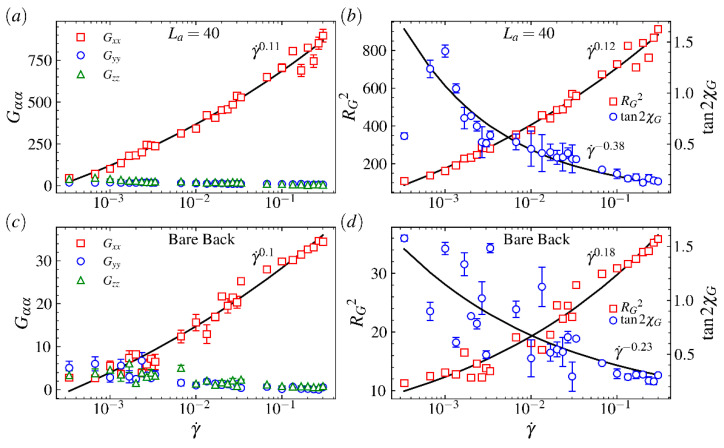
(**a**,**c**) Components in the gyration tensor ($G_{xx}$, $G_{yy}$, $G_{zz}$) and (**b**,**d**) squared radius of gyration tensor $R_{G}{}^{2}$ and alignment parameter $\tan2\chi_{G}$ as a function of Weissenberg number Wi for arm length (**a**,**b**) $L_{a}=0$ (bare backbone) and (**c**,**d**) $L_{a}=40$.

**Figure 10:**
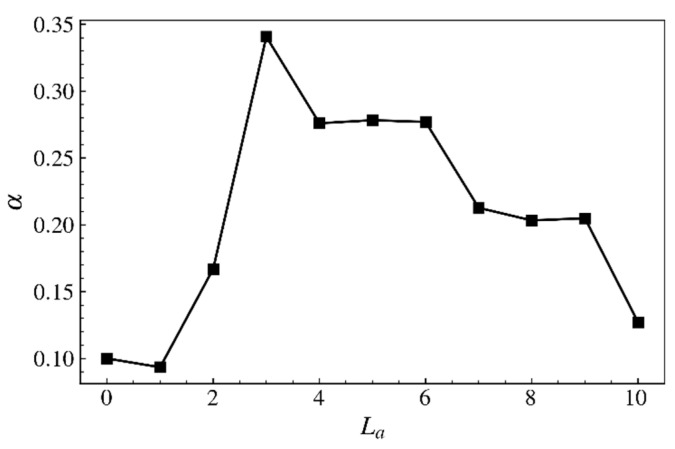
The exponent of power-law relation $G_{xx}∼{\dot{\gamma }}^{\alpha }$ (see Figure 9) for various comb polymers as a function of the arm length.

To characterize the conformational properties of the comb polymer in shear flow, we evaluate the average gyration tensor

(2) $$G_{\alpha \beta }=\frac{1}{N}\sum_{i=1}^{N}\langle r_{i,\alpha }r_{i,\beta }\rangle ,$$

where $N=L_{b}+N_{a}L_{a}$ is the total number of beads on a comb polymer, $r_{i,\alpha }$ is the position of bead *i* relative to the center of mass of the comb polymer, and α,β∈{x,y,z}. The alignment of a comb polymer is characterized by the orientation angle $\chi_{G}$. In our simulation system, the flow direction is along *x*-axis and the velocity-gradient direction is along *z*-axis. Then the orientation angle $\chi_{G}$ can be determined by

(3) $$\tan(2\chi_{G})=\frac{2G_{xz}}{G_{xx}-G_{zz}}\equiv m_{G}/Wi,$$

where the orientation resistance parameter $m_{G}$ is defined by the right side of equation [7,8]. The components in the average gyration tensor of comb polymers as a function of $\dot{\gamma }$ are plotted in Figure 9a. The squared radius of gyration tensor $R_{G}{}^{2}$ and alignment parameter $\tan(2\chi_{G})$ vs. $\dot{\gamma }$ are plotted in Figure 9b. To explore the impacts of the arm impose on the backbone, the same analysis on gyration tensor for a bare backbone are plotted in Figure 9c,d. As shown in Figure 9a,c, $G_{xx}$ increases largely while $G_{yy}$ and $G_{zz}$ decrease slightly as the shear rate $\dot{\gamma }$ increases. The increment of $G_{xx}$ for $L_{a}=40$ is much larger than that for the bare backbone ($L_{a}=0$) because long arms expand the comb polymer’s configuration greatly. However, a power-law fit yields the dependence $G_{xx}∼{\dot{\gamma }}^{0.11}$ for the comb polymer and $G_{xx}∼{\dot{\gamma }}^{0.1}$ for the bare backbone. The similar dependences indicate that the comb polymer with very long arms is at the same level of sensitivity to shear flow as the bare backbone and configurations of these two types of polymers will change slowly as the shear rate increases in medium and strong shear flow regime. However, the exponent of the dependence for the comb polymer with the arm length between 0 and 40 increases to 0.3 and then decreases to a value about 0.1 (see Figure 10), indicating the bare backbone and comb polymer with very long arms are more vulnerable to stretching along the flow direction on exposure to shear flow. In fact, if the data can be collected from large enough shear flow regime, all exponents will decay to 0. It is because the configuration expansion is close to the limit when the shear rate grows further from a high value that such a result can be easily inferred from the changes in the backbone’s length at the fully stretched state shown in Figure 6a. As shown in Figure 9b,d, a power-law fit yields the dependence $R_{G}{}^{2}∼{\dot{\gamma }}^{0.12}$ for the comb polymer with arm length 20 and $R_{G}{}^{2}∼{\dot{\gamma }}^{0.18}$ for the bare backbone. These results indicate the configuration of the comb polymer with long arms is more compact than the bare backbone and the structure of the comb polymer is more steady in strong shear flow regime. Moreover, Figure 9b,d show the dependence $\tan2\chi_{G}∼{\dot{\gamma }}^{-0.38}$ for the comb polymer and $\tan2\chi_{G}∼{\dot{\gamma }}^{-0.23}$ for the bare backbone, indicating that the resistance on the change in orientation for the comb polymer is stronger than the bare backbone when a shear field is introduced. 

**Figure 11:**
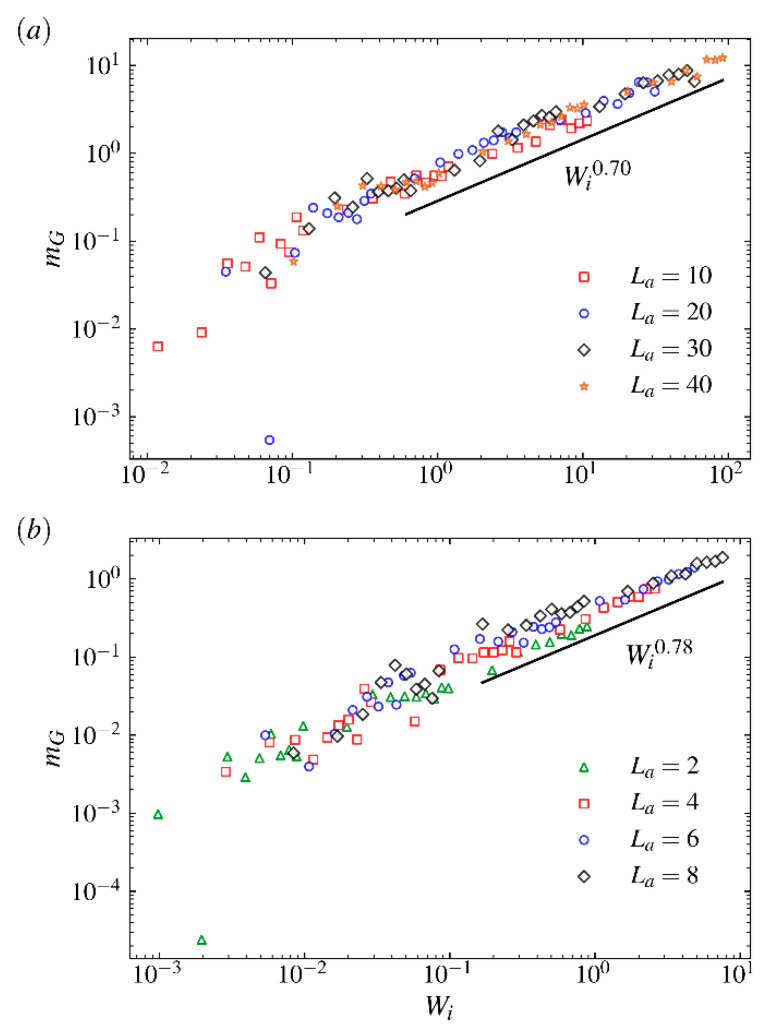
Orientational resistance parameter $m_{G}$ as a function of the Weissenberg number Wi for comb polymers with (**a**) long arms and (**b**) short arms.

Figure 11 shows the orientational resistance parameter $m_{G}$ as a function of Wi for comb polymers with long arms (a) and short arms (b). A power-law relation, i.e., $m_{G}∼f^{\alpha }Wi^{\mu }$, of which $m_{G}$ for star polymers with f arms in shear flow under large Wi was found in [5]. We also evaluate our simulations to fit the power-law dependence of $m_{G}$ on Wi. The results are shown in Figure 11a,b. We obtain μ=0.78, which is also larger than the exponent μ=0.54±0.03 obtained for self-avoiding linear polymer [7,8] for SCB polymers. We obtain μ=0.70, which is larger than the exponent μ=0.65±0.03 obtained for star polymer [5] for LCB polymers. These differences show that there is great variation in conformational and dynamical properties between branched polymers with simple and complex structures. The decline in exponent for long arms indicates that LCB polymers with very long arms are vulnerable, in terms of their linear counterparts, to stretch along the flow direction on exposure to shear flow.

**Figure 12:**
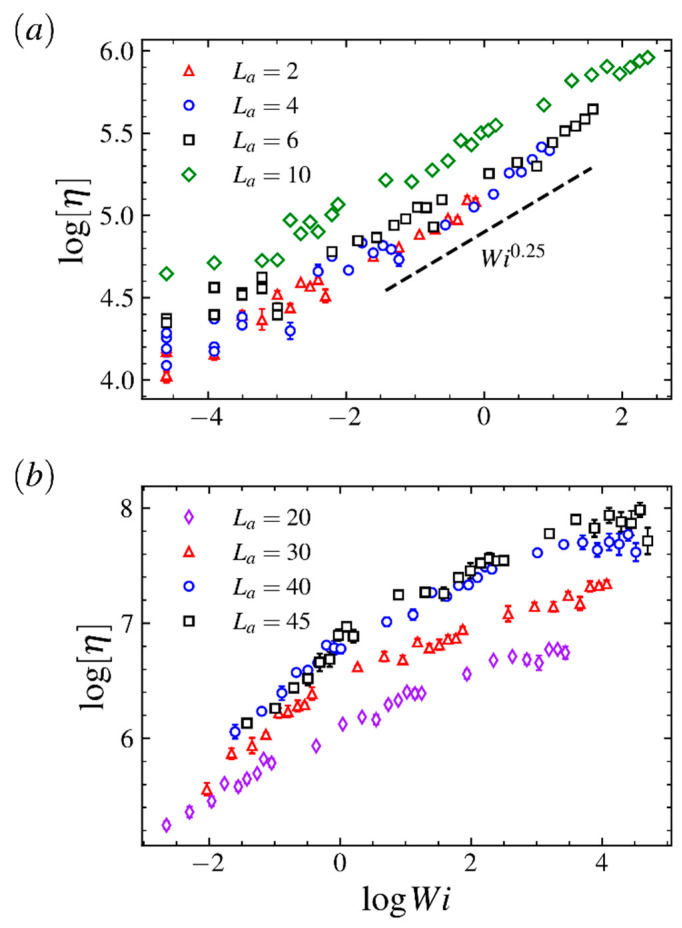
Intrinsic viscosity [η] as a function of the Weissenberg number Wi for comb polymers with (**a**) long arms and (**b**) short arms.

To measure the LCB polymer’s ability to impact the shear flow, we evaluate the intrinsic viscosity [η] for various arm length and shear flow strength. The intrinsic viscosity [η] is determined by

(4) $$\left[\eta \right]=\underset{c\to 0}{\lim}\frac{{\dot{w}}_{1}+{\dot{w}}_{2}}{\eta_{0}{\dot{\gamma }}^{2}c},$$

where $\eta_{0}$ is the viscosity of the solvent, *c* is the concentration, ${\dot{w}}_{1}$ is the rate of energy dissipation due to the perturbation of flow field and ${\dot{w}}_{2}$ is the frictional dissipation due to the rotation of the polymer [56]. Figure 12 shows the log-log plot of the intrinsic viscosity vs. Wi for comb polymers with various arm lengths. As shown in Figure 12a, for the comb polymer with shorter arms ($L_{a}\leq 10$), the dependence of the intrinsic viscosity on Wi reads $\left[\eta \right]∼Wi^{0.25}$. The consistent results for various arm lengths indicate that the gradual increasing in arm length in the short arm regime will not dramatically change the comb polymer’s ability and mechanism in changing the property of shear flow. However, as shown in Figure 12b, the power-law relation decays in the long arm and strong flow regime, indicating that properties in the comb polymer with very long arms are quite distinct from those with shorter arms. Such results are in accordance with the dynamical properties in LCB polymers discussed before.

## 3. Materials and Methods

### 3.1. Comb Polymer

**Figure 13:**
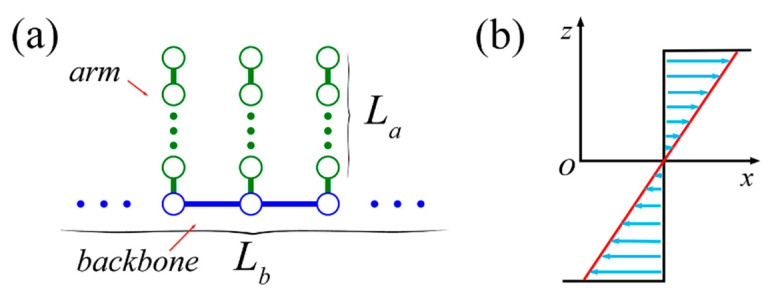
(**a**) Schematic of the LCB comb polymer with backbone length $L_{b}$ and arm length $L_{a}$. (**b**) The profile of shear flow in *xoz* view.

In our model system, a comb polymer is composed of many evenly distributed linear arms and a linear backbone in which each monomer is linked by one end of an arm (see Figure 13a). The monomer number of the backbone and each arm are $L_{b}$ and $L_{a}$, respectively. The arm number of the polymer is $N_{a}$. Correspondingly, the total monomer number is $N=L_{b}+N_{a}L_{a}$. Specifically, the monomers of the backbone and arms having excluded-volume short-range interactions between non-bonded beads are taken into account by the Lennard–Jones (LJ) potential

(5) $$U_{LJ}\left(r\right)=\{\begin{array}{ll} 4\varepsilon \left[{\left(\frac{\sigma }{r}\right)}^{12}-{\left(\frac{\sigma }{r}\right)}^{6}\right], & r\leq 2^{1/6}\sigma \\ 0, & r>2^{1/6}\sigma \; \end{array},$$

where $r=\left|r_{i}-r_{j}\right|$ denotes the distance between the centers of two monomers *i* and *j* located at $r_{i}$ and $r_{j}$. σ and ε are taken as unit length and energy, respectively.

The bond that connects adjective monomers is described by a finitely extensible nonlinear elastic (FENE) potential [57]

(6) $$U_{FENE}\left(r\right)=-\frac{Kl_{0}^{2}}{2}\ln\left[1-{\left(\frac{r}{l_{0}}\right)}^{2}\right],r<l_{0},$$

where r denotes the distance between the centers of two monomers connected by the bond, $l_{0}$ is the maximum bond length and *K* is the spring constant. $l_{0}=1.5\sigma$ is chosen to avoid bond crossing [58] and the spring constant is chosen to be $K=30\varepsilon /\sigma^{2}$.

### 3.2. Multiparticle Collision Dynamics (MPCD)

The explicit solvents are modeled as point-like particles of mass *m* in MPCD [59,60,61]. The dynamics proceeds in discrete time increments *h*, denoted as collision time, by alternating streaming and collision steps [61,62]. In the streaming step, the solvent particles move ballistically with their respective velocities within the time interval *h* between two consecutive collision steps and the position of solvent particle *i* is updated according to

(7) $$r_{i}(t+h)=r_{i}(t)+v_{i}(t)h.$$

In the collision step, all the particles are sorted into cubic cells of edge length *a*. Their relative velocities with respect to the center-of-mass velocity of the cell which they locate are rotated around a randomly oriented axis by a fixed angle α [63]. The velocity of solute *i* after the collision step is updated according to

(8) $$v_{i}(t+h)=v_{cm}(t)+(R(\alpha )-I)[v_{i}(t)-v_{cm}(t)],$$

where R(α) is the rotation matrix determined by α, I is the unit matrix and $v_{cm}=\sum_{j=1}^{N_{c}}v_{j}/N_{c}$ is the center-of-mass velocity of the cell with $N_{c}$ solute particles for pure fluid simulations. Mass, energy and momentum are conserved in this process, which ensures that hydrodynamic behavior emerges on larger-length scales [61,64]. 

The coupling of comb polymers and the fluid occurs in the collision step; here, each cell contains $N_{{}_{c}}^{s}$ solute particles and $N_{{}_{c}}^{m}$ monomers of polymer; thus, the center-of-mass velocity of the cell at time *t* should be determined by [65,66]

(9) $$v_{cm}(t)=\frac{\sum_{i=1}^{N_{c}^{s}}mv_{i}(t)+\sum_{j=1}^{N_{c}^{m}}Mv_{j}(t)}{mN_{c}^{s}+MN_{c}^{m}}.$$

The mass, local momentum and energy are still conserved during the collision step. To ensure the Galilean invariance, a random shift in the collision grid is performed before every collision step [67,68].

Lees–Edwards boundary conditions are applied to generate shear flow [69]. This yields a linear fluid velocity profile $v=\dot{\gamma }z\hat{x}$ along the flow direction (*x*-axis). A local cell-based Maxwell–Boltzmann thermostat is applied by which velocities are scaled to maintain the desired temperature of the system [70].

### 3.3. Simulation Details

We simulate the comb LCB polymer immersed in steady shear flow, as shown in Figure 11b, by means of hybrid method which combines molecular dynamics (MD) for the comb polymer and MPCD for the solvent particles. 

For the fluid, we take the parameters that the time unit $\tau =\sqrt{ma^{2}/k_{B}T}$, the collision period h=0.1τ, the cubic cell length a=σ, the rotation angle $\alpha =130^{\circ }$ and the average number of fluid particles in a collision cell $\langle N_{c}^{s}\rangle =10$, which yields the solvent viscosity $\eta_{s}=8.7\sqrt{mk_{B}T/a^{4}}$ which has been shown to be a reliable parameter to account for liquid-like properties [71]. For the polymer, we set its monomer mass  M=10m and use the velocity–verlet algorithm with time step $h_{p}=0.005\tau$ to integrate Newton’s equations of motion. The relaxation behavior of an LCB polymer can be characterized by the exponential decay of the autocorrelation function $C_{ete}(t)$ of the end-to-end vector of arms,

(10) $$C_{ete}(t)=\frac{<R_{ete}(t)R_{ete}(0)>}{<R_{ete}(0)R_{ete}(0)>}=C_{0}\exp(-t/\tau_{eq}),$$

here, <⋯> means the average for all arms. In our simulation, a power-law fit yields the dependence $\tau_{eq}∼L_{a}^{1.55}$ for both LCB and SCB polymers. The strength of the shear flow is either characterized by the shear rate $\dot{\gamma }$ or the Weissenberg number $W_{i}=\dot{\gamma }\tau_{eq}$.

Each simulation runs in the cubic box with side length L=100σ and the periodic conditions are applied. For more accurate statistics, several independent runs were conducted for each set of parameters.

## 4. Conclusions

In this work, we investigate the effects of long arms and shear rates on the dynamical and structural properties for the LCB polymer using a hybrid simulation method. We analyze the dynamical properties by tracing the end-to-end distance of the backbones and arms. In addition, by tracking the variation in angles that the backbones make with respect to flow direction, we find that the tumbling behaviors of backbones are at a constant frequency at a large-time-scale simulation for various shear rates. By fitting the angles of the backbone along the trajectory to a periodic function, one can obtain the period for the tumbling and rotation motion of the LCB polymer. Through the analysis of tumbling behaviors and rotation motions along the simulation trajectory, we find that the backbone experiences two stretched–folded–stretched transitions during the period of each tumbling motion. The very steady and smooth transition on dynamical properties of LCB polymers is observed at medium shear flow strength, which means that the interactions among long arms and random motions of arms cannot compete with the force imposed by shear flow. By analyzing the rotation period of LCB polymers, we find that arms of branched polymer have a function in keeping the LCB polymer’s motion stable. Meanwhile, we find that the rotation period of the LCB polymer is mainly determined by the conformational distribution and the non-shrinkable state of structure along the velocity-gradient direction. Moreover, by analyzing the characteristic frequency of the tumbling motion, we also find that the comb polymer in shear flows change from linear-polymer-like to capsule-like behavior as the arm length increases.

To evaluate the effects of long arms on structures of LCB polymers for various Weissenberg numbers numerically, we carry out statistics on the gyration tensor. We find the $G_{xx}$, which is the component along the flow direction, increases dramatically as the shear rate increases. By exploring the relation between $G_{xx}$ and shear rate, we find that, as the arm length increases, the LCB polymer is more vulnerable to stretching along flow direction as the linear counterparts. By analyzing orientation resistance parameter $m_{G}$ and fitting it to a power-law dependence on Wi, we obtain μ=0.78 for shorter arms and μ=0.70 for longer arms. The results are very different from the linear and star counterparts, indicating that the random and fast motions of arms put very complex constraints on the motion of the comb polymer. The decline in the exponent also shows that the LCB polymer, like its linear counterpart, is vulnerable to stretching along the flow direction on exposure to shear flow. Our results may help to understand the dynamical and structural properties in the LCB polymers, which have a high density of arms in shear flow.
